# Refeeding Syndrome in Older Hospitalized Patients: Incidence, Management, and Outcomes

**DOI:** 10.3390/nu15184084

**Published:** 2023-09-21

**Authors:** Kevin Terlisten, Rainer Wirth, Diana Daubert, Maryam Pourhassan

**Affiliations:** Department of Geriatric Medicine, Marien Hospital Herne, Ruhr-Universität Bochum, 44625 Herne, Germany; k-terlisten@hotmail.de (K.T.); rainer.wirth@elisabethgruppe.de (R.W.); diana.daubert@elisabethgruppe.de (D.D.)

**Keywords:** malnutrition, refeeding syndrome, incidence, management, outcomes, older hospitalized patients

## Abstract

Refeeding syndrome (RFS) is a serious metabolic disturbance that manifests after reintroducing nutrition to severely malnourished individuals. Especially susceptible are older patients, due to higher malnutrition rates, although the incidence remains uncertain. Our study aimed to assess the occurrence and management of RFS in malnourished older hospitalized patients. This prospective study included 156 malnourished older patients, with malnutrition identified using the Mini Nutritional Assessment-Short Form. We evaluated critical biochemical parameters at admission and for ten days after starting nutritional therapy. Using the consensus evidence-based approach, we managed and evaluated RFS. We also tracked mortality and unexpected hospital readmissions for six months after discharge. The average patient age was 82.3 ± 7.5 years, with 69% female. Patients showed hypophosphatemia (23%), hypomagnesemia (31%), and hypokalemia (6%) on admission. Prior to nutritional replenishment, patients were classified as being at low (64%), high (30%), or very high risk (6%) for RFS. After nutritional therapy, 14% and 5% developed imminent and manifest RFS, respectively. There were no significant differences in six-month post-discharge mortality rates or unexpected hospital readmissions between patients with or without RFS. Despite adherence to guideline-recommended management, RFS can persist. No elevated mortality was noted in RFS patients, potentially due to early diagnosis and treatment.

## 1. Introduction

Refeeding syndrome (RFS) is recognized as a severe metabolic condition that manifests following the reintroduction of feeding—whether oral, enteral, or parenteral—in starved or severely malnourished individuals [1,2]. This complex clinical phenomenon is characterized by acute fluid and electrolyte disturbances, particularly affecting phosphate, potassium, magnesium, and sodium levels, often accompanied by thiamin deficiency [3,4]. If not promptly identified and appropriately managed, RFS can pose a life-threatening risk [5]. Clinical manifestations of RFS vary greatly, spanning from mild cases with minimal clinical signs and almost negligible patient risk, to severe cases resulting in clinical deterioration and even leading to sudden cardiac death [5,6,7]. The syndrome typically presents with a multitude of non-specific symptoms that often manifest 2–5 days post-initiation of nutritional therapy [3,4].

The incidence of RFS is likely to be substantial among older individuals due to the high prevalence of malnutrition in this population [8,9]. Our previous research underscored a significant correlation between malnutrition and RFS, where approximately 75% of the hospitalized elderly patients who were at malnutrition risk also presented a potential risk for developing RFS according to generally accepted risk factors [8]. However, it is worth noting that despite its frequent occurrence, the risk of RFS frequently goes undetected among older hospitalized patients even when signs of malnutrition are apparent. Therefore, it is not surprising that the incidence of RFS is unknown among these patients; however, it is suggested to be substantial.

There is currently no standardized, evidence-based guideline that unifies the definition and treatment approach for RFS. However, it is widely accepted that an effective risk assessment strategy, the development of a robust nutritional care plan, and monitoring of patients at risk of RFS could substantially reduce the morbidity and mortality associated with the syndrome [5]. Indeed, timely and precise monitoring and supplementation of serum electrolytes, notably magnesium and phosphate, represent key strategies in the early prevention and treatment of RFS. Additionally, measuring and supplementing thiamine, together with a cautiously designed nutritional regimen that initiates with low caloric intake, progressively elevated over 5–10 days based on individual risk for RFS and clinical presentation, serve as critical preventive and therapeutic measures for the syndrome [10,11,12].

Despite the National Institute for Health and Care Excellence (NICE) providing widely accepted guidelines for adult nutritional medical support [13,14], there is a gap in the management of RFS. While these guidelines do address the recognition of malnutrition and management of nutritional therapy, there is a growing need for more focused approaches to RFS prevention and treatment. A previously published expert consensus statement, built upon the review of RFS by Friedli et al., proposes a procedural approach for patient care during nutritional therapy to effectively prevent and manage RFS [5]. To date, there has been a scarcity of systematic studies investigating the incidence and treatment of RFS in geriatric patients. This present study therefore seeks to address this gap by exploring the occurrence and management of RFS in older hospitalized patients, utilizing the consensus-supported algorithm proposed by Friedli et al.

## 2. Materials and Methods

This research was structured as a prospective, observational study and performed at the geriatric acute care unit of Marien Hospital Herne, university hospital of Ruhr-University Bochum, Germany. This work is a subsection of a larger intervention study, focused on improving treatment protocols for malnutrition in older hospitalized patients. Within the scope of this larger investigation, proactive steps for RFS prevention, early detection, and treatment were also implemented. The study population comprised 156 malnourished older patients, who were consecutively admitted from May 2019 to October 2020. Patients were eligible for recruitment if they met the following criteria: malnutrition as defined by a Mini Nutritional Assessment Short Form (MNA-SF) score of less than 8 or a weight loss greater than 10% of initial body weight within the past six months, an age of 65 years or above, the necessity for nutritional support, an anticipated hospital stay in the geriatric department of a minimum of 14 days, and the ability and willingness to provide informed consent. Exclusion criteria included severe dementia, dysphagia and depression, anticipated requirement for tube feeding for a period exceeding two weeks, a palliative situation, and severe cognitive impairment. Data of the study were managed using the REDCap electronic data capture tool [15], hosted at Marien Hospital Herne. This secure, web-based software provided an efficient and reliable means of collecting and storing participant data, ensuring accuracy and consistency throughout the study.

### 2.1. Geriatric Assessment

Within the first few days following hospital admission, all participants underwent a comprehensive geriatric assessment. Nutritional status was evaluated using the MNA-SF, which assesses reduction in food intake, WL during last 3 months, mobility, psychological stress and acute diseases, neuropsychological problems, and BMI [16]. The resulting scores categorized patients into three groups: those with normal nutritional status (12–14 points), those at risk of malnutrition (8–11 points), and those classified as malnourished (0–7 points). Functional ability related to activities of daily living was assessed through the German version of the Barthel Index (BI) [17]. The index, on a scale of 0 to 100 points, considers a score of 100 as indicative of complete independence. The Frail Simple scale was employed to assess frailty, with scores of 1–2 and 3–5 signifying pre-frailty and frailty, respectively [18]. In addition, sarcopenia risk was assessed using the SARC-F questionnaire (Strength, Assistance in walking, Rise from a chair, Climb stairs, and Falls), which utilizes a 0–10 scale. A score exceeding 4 was suggestive of probable sarcopenia [19,20]. Depressive symptoms were examined through the Depression in Old Age Scale (DIA-S), which allowed for classification into three categories: no depression (0–2 points), suspected depression (3 points), and probable depression (4–10 points) [21]. Cognitive status was evaluated using the Montreal Cognitive Assessment (MoCA) [22], where a score below 26 was considered indicative of cognitive impairment. Furthermore, the Parker Mobility Score was utilized to evaluate patients’ mobility status. This scoring system consists of three mobility-focused queries, each with a scoring range of 0 to 3. The total score, obtained by summing the individual assessments pertaining to the patient’s ability to navigate within the house, step outside the house, and manage shopping tasks, can vary from 0 to 9. These specific tasks are graded based on the ability to perform without struggle (3 points), reliance on assistive devices (2 points), dependency on human aid (1 point), or a complete incapacity to carry out the task (0 points). A perfect score of 9 signifies that an individual possesses optimal mobility [23]. In addition, nutritional support was provided to all malnourished patients. The nutritional support extended to patients primarily consisted of oral nutrition supplements and protein shakes. Specifically, each 100 mL serving of the protein shake provided 140 kcal, 11.4 g of protein, 15.5 g of carbohydrate, and 4.5 g of fat.

### 2.2. Laboratory Parameters

Blood samples were obtained and measured according to standard clinical procedure. Serum levels of key biochemical parameters including phosphate, potassium, magnesium, sodium, calcium, glucose, urea, and creatinine were analyzed on admission and subsequently on the 1st, 2nd, 3rd, 5th, 7th, and 10th days after the initiation of nutritional therapy. The reference ranges for these parameters, in the context of a healthy population, are presented in Table 2. Due to a transition between laboratories during the study, vitamin B1 measurements were conducted using the initial method for the initial group of patients, and a different method was employed for subsequent patients in the new laboratory.

### 2.3. Refeeding Syndrome

The risk of developing RFS was evaluated upon admission, utilizing an evidence-based approach proposed by Friedli et al. [5]. According to this methodology, patients exhibiting at least one of the following minor parameters (BMI < 18.5 kg/m^2^, unintentional weight loss > 10% in the preceding 3–6 months, or no nutritional intake for >5 days or a medical history of alcohol or drug abuse), or one of the major risk factors (BMI < 16 kg/m^2^, unintentional weight loss > 15% in the preceding 3–6 months, or no nutritional intake for >10 days, or pre-existing low levels of potassium, phosphate, or magnesium prior to refeeding), or one of the very high risk features (BMI < 14 kg/m^2^, weight loss > 20%, no nutritional intake for >20 days) were classified as minor, major, or very high risk for RFS, respectively. For patients identified to be at risk of RFS, appropriate therapeutic measures were implemented to prevent the onset of the syndrome as proposed by Friedli et al. [5]. In addition, all patients at risk of RFS were provided with thiamine substitution based on the protocol. According to Friedli et al., imminent RFS was diagnosed if, within 72 h of starting nutritional therapy, there was a reduction in phosphate levels from baseline by >30% or to less than 1.86 mg/dL, or a concomitant shift in two other electrolytes below the normal range (magnesium < 1.82 mg/dL, phosphate < 2.48 mg/dL, potassium < 3.5 mmol/L). Manifest RFS was diagnosed if the aforementioned electrolyte imbalances were accompanied by typical clinical symptoms such as peripheral edema, physical weakness, apathy, disorientation, and tachycardia. All malnourished patients were provided with oral nutrition supplements (ONSs), beginning with a low-energy intake (300 kcal) which increased gradually according to the patients’ requirements and preferences.

### 2.4. Follow-Up Examination

During follow-up, we investigated the incidence of adverse events, including instances of infections, unexpected hospital re-admissions, and cases of mortality. These incidents were documented during the patient’s hospitalization and then followed up at three and six months after discharge via standardized brief telephone interviews.

### 2.5. Statistical Analysis

The data were analyzed utilizing the SPSS statistical software package (SPSS Statistics for Windows, IBM Corp, Version 29.0, Armonk, NY, USA). For variables with a normal distribution, we represented continuous data via mean values and standard deviations (SDs). For data not normally distributed, median values alongside interquartile ranges (IQRs) were displayed. Categorical variables were presented as count totals and proportions (n, %). The independent sample t-test was used to compare normally distributed data, while the Mann–Whitney U test was employed for non-normally distributed data. Categorical data comparison was executed through the Chi-square test or Fisher’s exact test, as per the appropriateness. A *p*-value of less than 0.05 was set as the threshold for statistical significance.

## 3. Results

Baseline characteristics of study participants are presented in Table 1. The study population consisted of 156 participants with an age range of 64–100 years (69% females). Major reasons for hospital admission included cardiovascular diseases, falls, fractures, post-stroke care, urinary tract infections, and primary neurodegenerative diseases. The BMI ranged from 10.7 to 40.6 kg/m^2^, with 2% and 10% of patients having a BMI below 16 kg/m^2^ and 18.5 kg/m^2^, respectively. All patients were malnourished, with a median MNA-SF score of six. Although eight patients were initially categorized as being at risk of malnutrition, their significant unintentional weight loss (>10% within six months) classified them as malnourished. Moreover, 97% reported previous unintentional weight loss, with a mean weight loss of 11.5 ± 7.1 kg within an average duration of 8 months (18.3% decrease from the initial mean body weight). Frailty and cognitive impairment were predominant in the cohort, with 90% (n = 140) frail and 93% (n = 145) having impaired cognitive function. SARC-F indicated probable sarcopenia in 89% (n = 138). Based on the DIA-S, 42% (n = 65) exhibited no depressive symptoms, while 16% (n = 25) and 41% (n = 63) had suspected and probable depression, respectively. Clinical assessment using the DIA-S was not possible for three patients due to language barriers, and their data are consequently absent from the related findings. At hospital admission, bacterial and viral infections were present in 21% and 7% of patients, respectively. Nutritional support was provided to all malnourished patients, with 2% (n = 3) refusing it.

Mean serum concentrations of laboratory data are given in Table 2. Upon admission, the observed prevalence for hypophosphatemia, hypomagnesemia, and hypokalemia was 23% (n = 36), 31% (n = 48), and 6% (n = 9), respectively. The diagnostic procedure for initial risk assessment and identification of RFS is given in Table 3. Prior to nutritional replenishment, 64% (n = 100), 30% (n = 46), and 6% (n = 10) of patients were classified as having low, high, and very high risk of RFS, respectively. The observed reductions in the levels of phosphate, magnesium, and potassium post-initiation of nutritional therapy (within the first 72 h) were in 4% (n = 6), 56% (n = 87), and 17% (n = 26) of the patients, respectively (see Table 3 and Methods Section, refeeding syndrome). This was in addition to those who presented with low levels upon their admission. Notably, a concurrent decrease in any two other electrolytes was observed in 17% (n = 27) of patients.

**Table 2:** Mean serum concentrations of laboratory data on admission.

	Total Population (n = 156)	Reference Range
Phosphate (mg/dL)	3.1 ± 0.6	2.7–4.5
Low (n; %)	36 (23)	
Normal (n; %)	116 (76)	
High (n; %)	1 (1)	
Magnesium (mg/dL)	1.8 ± 0.3	1.7–2.55
Low (n; %)	48 (31)	
Normal (n; %)	106 (69)	
High (n; %)	0 (0)	
Potassium (mmol/L)	4.2 ± 0.6	3.3–5.1
Low (n; %)	9 (6)	
Normal (n; %)	135 (88)	
High (n; %)	10 (6)	
Sodium (mmol/L)	139.1 ± 4.3	136–145
Low (n; %)	24 (16)	
Normal (n; %)	126 (81)	
High (n; %)	5 (3)	
Calcium (mmol/L)	2.2 ± 0.1	2.15–2.55
Low (n; %)	58 (38)	
Normal (n; %)	95 (61)	
High (n; %)	2 (1)	
Glucose (mg/dL)	122.5 ± 55.7	74–106
Low (n; %)	23 (15)	
Normal (n; %)	74 (48)	
High (n; %)	57 (37)	
Urea (mg/dL)	40.5 ± 21.5	21–43
Low (n; %)	22 (14)	
Normal (n; %)	80 (52)	
High (n; %)	52 (34)	
Creatinine (mg/dL)	1.0 ± 0.4	0.5–1.0
Low (n; %)	4 (3)	
Normal (n; %)	99 (58)	
High (n; %)	60 (39)	
* Vitamin B1 (ng/mL)	72.6 ± 34.7	20–100
Low (n; %)	0 (0)	
Normal (n; %)	49 (86)	
High (n; %)	8 (14)	
* Vitamin B1 (nmol/L)	111.9 ± 41.1	70–180
Low (n; %)	7 (7)	
Normal (n; %)	80 (86)	
High (n; %)	6 (7)	

Values are given as mean ± SD or number (%). * Due to the laboratory change during the study, vitamin B1 measurements were conducted using the initial method for the initial group of patients, and a different method was employed for subsequent patients in the new laboratory.

From the total population, 81% (n = 126) did not exhibit signs of RFS, whereas imminent and manifest RFS were detected in 14% (n = 22) and 5% (n = 7) of patients, respectively (Table 3). Examining the subgroup that was diagnosed with manifest RFS more closely, we found that 57% (n = 4) of these patients were severely malnourished, with MNA-SF scores ≤ 4, and 86% (n = 6) reported an average prior unintentional weight loss of 11.8 ± 4.4 kg over a mean duration of 4.3 ± 2.0 months. In addition, several clinical symptoms after beginning nutritional therapy were observed. The majority (n = 5, 71%) exhibited physical weakness, while apathy and tachycardia were noticed in two (29%) of these patients. It is important to note that while many older patients exhibit weakness upon admission, our emphasis was on those whose weakness newly manifested and coincided with disturbances in phosphate or other relevant electrolytes after starting nutritional therapy. Remarkably, three (43%) patients who developed manifest RFS were initially categorized as being at high or very high risk for RFS upon admission.

At six months post-discharge, the recorded mortality rate amounted to 17% of the total study population, encompassing 26 patients. Moreover, unexpected hospital readmissions were documented for 45% (n = 58) of the participants. The data revealed no substantial differences in mortality rates (*p* = 0.584) and unexpected hospital readmissions (*p* = 0.192) between patients diagnosed with imminent or manifest RFS and those without. In a comparison focusing solely on patients exhibiting manifest RFS versus those without RFS, no significant differences in mortality rates (*p* = 0.594) and unexpected hospital readmissions (*p* = 0.366) were apparent.

## 4. Discussion

Our study delivers important insights into the incidence and management of RFS among older hospitalized patients within the setting of geriatric acute care. Despite the increasing recognition of RFS in recent years, data specific to its prevalence and management strategies in geriatric populations remain notably limited. Our research works towards bridging this knowledge gap by systematically evaluating the incidence, risk factors, and associated outcomes of RFS in a cohort of malnourished older hospitalized patients. Our findings emphasize the efficacy of integrating early detection and prevention strategies for RFS into malnutrition treatment protocols for mitigating severe outcomes.

Many hospitals have integrated the early initiation of nutritional support into their standard of care to prevent the associated morbidity burden of disease-induced malnutrition [24,25]. The consensus-supported algorithm proposed by Friedli et al. [5] and the NICE guidelines [13] have identified key risk factors for RFS, encompassing low BMI, unintended weight loss, negligible or non-existent nutritional intake, alcohol abuse, and diminished admission levels of certain electrolytes. Our research substantiates these identified risk factors, with our patient demographic showing 64% at low risk for RFS, 30% at high risk, and an alarming 6% at very high risk on admission. These data illuminate the increased vulnerability of older hospitalized patients to this syndrome, confirming our earlier research indicating that approximately three-quarters of hospitalized geriatric patients at risk of malnutrition also exhibited a substantial risk of RFS [8]. The substantial prevalence of risk factors identified in our current research also aligns with a previous study conducted on internal medicine patients [26]. In this prior study, out of 178 participants, 97 patients, representing 54%, were evaluated as being at risk of developing RFS. This percentage mirrors the current findings. Furthermore, the previous research revealed that out of these 97 patients, 14 patients (comprising 14% of those at risk and 8% of the overall study population) indeed developed the syndrome, underscoring the serious potential of RFS risk turning into actual cases. In the present study, we noted imminent and manifest RFS in 14% and 5% of the malnourished study population, respectively. In addition, nearly half (43%) of the patients who eventually developed manifest RFS were initially classified as being at high or very high risk for RFS upon admission. This highlights the persisting challenge posed by RFS even with early detection and preventive measures.

The results of our study align with previous research findings that emphasize the importance of early recognition and prevention of RFS [6,11,12] and confirm the utility of the consensus-supported algorithm proposed by Friedli et al. [5] for risk stratification and management of RFS. Nevertheless, our results underscore that even with these protocols in place, a proportion of patients still develop RFS, indicating the necessity for further optimization of preventive and therapeutic strategies. However, it is worth mentioning that the definition of RFS lacks universal acceptance, with most of the available evidence being limited. Current evidence supports the myriad benefits of prompt nutritional intervention in medical inpatients [25,27]. A systematic review by Matthews-Rensch et al. [28] examined 24 studies (one RCT and 23 observational), aiming to understand feeding methodologies and their impact on RFS outcomes. The findings showed varied feeding methods and no clear consensus on optimal feeding strategies to reduce RFS risk. Currently, there is no strong evidence suggesting that starting with higher-energy feedings has negative effects on those prone to RFS, highlighting the need for further research.

Another key finding from our study is the absence of significant differences in the six-month post-discharge mortality rates between patients who developed RFS and those who did not. This could suggest that early detection followed by effective management may substantially mitigate adverse outcomes typically associated with the syndrome. However, the absence of a significant difference might also be attributed to the small number of deaths recorded during the study period. Our study’s findings stand in contrast to those from a recent secondary analysis of the EFFORT trial which illustrated a significant correlation between RFS and long-term mortality in malnourished medical inpatients, along with other adverse clinical outcomes, such as escalated risk for ICU admission and protracted hospital stay [29]. On the other hand, another retrospective cohort study among 3854 hospitalized patients exhibited no notable correlation between RFS and increased mortality, a finding that aligns with our results. However, this study examined only short-term mortality of 30 days, and the definition criteria for RFS were based on the ASPEN guidelines (utilizing a lower threshold for RFS detection and not considering the presence of clinical symptoms), with 90% of all patients studied developing RFS [30,31]. It is worth noting that multiple factors contribute to mortality in geriatric populations. Older adults often present with a range of comorbidities, which may independently affect mortality, irrespective of RFS. This underlines the importance of comprehensive management approaches that address all potential risk factors to improve survival outcomes amongst older adults.

Some limitations to the present study should be discussed. First, this is a single-center study, and our findings may not be generalizable to other hospital settings, including hospitals with different patient demographics, care models, or protocols for the management of malnutrition and RFS. Second, our study relied on the consensus-supported algorithm proposed by Friedli et al. [5] for risk stratification of RFS. While this algorithm is based on recognized risk factors, it may not capture all the variables contributing to RFS, and its predictive value for RFS in different patient populations is not yet fully established. Third, given the nonspecific nature of RFS symptoms, in conjunction with the presence of multimorbidity and the inherent vulnerability of older patients, the accurate identification of RFS can sometimes be challenging, particularly within our cohort. Changes in unspecific clinical symptoms may occur coincidently but independently from RFS. Lastly, the relatively small number of deaths during the study period may have limited our ability to detect significant differences in mortality rates between patients who developed RFS and those who did not. Larger, multicenter studies would provide more definitive answers regarding the impact of RFS on mortality. While acknowledging these limitations, we believe our study makes a significant contribution to the understanding of RFS in older hospitalized patients, a population at high risk for malnutrition and its complications. Future research should aim to validate and refine risk stratification algorithms and to prospectively investigate the impact of RFS and its management on clinical outcomes in older patients.

## 5. Conclusions

Despite strict adherence to guideline-recommended management, the occurrence of RFS can still persist, as observed in our study. Interestingly, no increase in mortality was detected at six months post-discharge, which could be attributed to the successful early detection of the syndrome. Our study underscores the importance of early detection and careful management of RFS in older hospitalized patients, especially those with high risk factors. However, the persistence of RFS despite the implementation of current protocols underscores the need for further research to enhance prevention and treatment approaches. In addition, the association between RFS and long-term mortality remains a topic that warrants further investigation.

## Figures and Tables

**Table 1:** Characteristics of study population on admission.

	All (n = 156)
Gender (n, %)	
Females	108 (69)
Males	48 (31)
Age (y)	82.3 ± 7.5
Height (m)	1.64 ± 0.08
Body weight (kg)	62.8 ± 13.9
BMI (kg/m^2^)	23.1 ± 4.5
Geriatric assessments	
MNA-SF, Median (IQR)	6 (5–7)
At risk of malnutrition (n, %)	8 (5)
Malnourished (n, %)	148 (95)
Barthel-Index, Median (IQR)	45 (40–59)
Parker mobility score, Median (IQR)	3 (2–5)
Frail Simple score, Median (IQR)	4 (4–5)
SARC-F scores, Median (IQR)	7 (5–8)
Depression score (DIA-S), Median (IQR)	3 (1–5)
Cognitive function (MoCA), Median (IQR)	17 (13–21)
Handgrip strength, Median (IQR)	14.5 (8–20)
Anticoagulation (n, %)	
Yes	43 (28)
No	112 (72)
Weight loss	
Yes (n, %)	151 (97)
Unintentional weight loss in kg	11.5 ± 7.1
Unintentional weight loss within months	8.1 ± 7.1
No (n, %)	4 (3)
Nutrition therapy (n, %)	
Yes	153 (98)
No	3 (2)
Bacterial infections (n, %)	
Yes	33 (21)
No	121 (79)
Virus infections (n, %)	
Yes	10 (7)
No	140 (93)
Discharge to (n, %)	
Home	122 (80)
Short term-care	23 (15)
Long term-care	3 (2)
Rehabilitation clinic	1 (1)
Another hospital	5 (3)
Length of stay in days, Median (IQR)	19 (14–21)

MNA-SF, Mini Nutritional Assessment Short Form; SARC-F, Strength, Assistance in walking, Rise from a chair, Climb stairs, and Falls; DIA-S scores, Depression in Old Age Scale; MOCA, Montreal Cognitive Assessment; Values are given as number (%), mean ± SD, or median (IQR, interquartile range).

**Table 3:** Diagnostic work-up for identifying patients with imminent and manifest refeeding syndrome in total study population (n = 156).

Initial Risk Assessment	n (%)
Minor risk factors	100 (64)
Major risk factors	46 (30)
High risk factors	10 (6)
* During the first 72 h after the start of nutrition therapy	
Decrease in phosphate	6 (4)
Decrease in magnesium	87 (56)
Decrease in potassium	26 (17)
** Decrease in any 2 other electrolytes	27 (17)
Refeeding Syndrome	
No	126 (81)
Imminent without clinical symptoms	22 (14)
Manifest with clinical symptoms	7 (5)

* Please see methods section, refeeding syndrome; ** any 2 other electrolyte shifts below normal range (magnesium < 1.82 mg/dL, phosphate < 2.47 mg/dL and, potassium < 3.5 mmol/L).

## Data Availability

Additional data are available from the corresponding author upon reasonable request.
